# Measuring financial distress in German cancer patients: development and validation of the Financial Distress of Cancer Assessment Tool (FIAT)

**DOI:** 10.1016/j.esmoop.2024.103992

**Published:** 2024-11-29

**Authors:** L. Richter, S. Pauge, K. Mehlis, A. Zueger, B. Surmann, V. Mathies, W. Greiner, T. Ernst, E.C. Winkler, N. Menold

**Affiliations:** 1 Methods in Empirical Social Research, Institute of Sociology, Faculty of Arts, Humanities and Social Science, Dresden University of Technology, Dresden, Germany; 2 Department for Health Economics and Health Care Management, School of Public Health, Bielefeld University, Bielefeld, Germany; 3 National Center for Tumor Diseases (NCT), NCT Heidelberg, a partnership between DKFZ and Heidelberg University Hospital, Heidelberg University, Medical Faculty Heidelberg, Department of Medical Oncology, Section Translational Medical Ethics, Heidelberg, Germany; 4 Department of Hematology/Oncology, Clinic of Internal Medicine II, Jena University Hospital, Jena, Germany

**Keywords:** subjective financial distress, patient-reported outcome measure, cancer, oncology

## Abstract

**Background:**

Cancer diagnosis and therapy can lead to significant financial distress for those affected, even in universal health care systems. We present the development and validation of a patient-reported outcome measure for financial distress in German cancer patients.

**Methods:**

Validation of the newly developed instrument followed a two-step approach, including two quantitative paper–pencil surveys (*N1* = 111, *N2* = 267) with patients of all types of cancer and treatment status at two German university hospitals. Factorial validity, reliability, construct, and criterion validity were assessed using exploratory and confirmatory factor analysis, correlative and linear regression analysis.

**Results:**

The Financial Distress of Cancer Assessment Tool (FIAT) comprises 19 items across three domains of subjective financial distress: (i) financial worries; (ii) dissatisfaction across various life domains, and (iii) challenging experiences with authorities and benefit providers (e.g. employment agency, health insurance). Confirmatory factor analysis confirmed the instrument’s factorial structure. Composite reliability (Raykov’s rho) ranges from 0.88 to 0.96, and retest reliability ranges from 0.64 to 0.75. Correlational analyses showed significant associations between FIAT scores and related constructs [e.g. correlations with the EORTC-QLQ-C30 financial distress subscale (Q28) ranging from 0.47 to 0.60], supporting its construct validity. Additionally, higher FIAT scores were significantly associated with lower health-related quality of life measured by Q29 and Q30 of the EORTC-QLQ-C30, with correlations ranging from −0.21 to −0.28. They were also positively correlated with depression (PHQ-4), with correlations ranging from 0.33 to 0.45, and anxiety (PHQ-4) with correlations ranging from 0.25 to 0.36, confirming its criterion validity.

**Conclusions:**

The newly developed patient-reported outcome measure is the first reported measurement tool to assess financial distress in German cancer patients. The instrument can be used for research purposes and to enable the provision of coordinated support services.

## Highlights

• We developed and validated the first patient-reported outcome measure (PROM) for financial distress in German cancer patients.
• The PROM assesses financial worries, life dissatisfaction, and challenges in navigating the health and welfare system.
• The PROM demonstrated strong psychometric properties including reliability and factorial, construct, and criterion validity.

## Introduction

Financial burden due to cancer can lead to psychosocial consequences for those affected, such as the development of depression, anxiety, and deterioration in quality of life (QoL).^1^, ^2^, ^3^, ^4^ A recent review by Longo et al.^5^ demonstrated that patients in publicly funded health care systems like Germany are also confronted with financial burdens due to cancer. The phenomenon will become even more relevant to be addressed by health care systems in the future, as cancer shifts towards a chronic disease and the number of cancer patients is steadily increasing due to demographic changes. Germany, for instance, faces nearly 500 000 new cancer cases annually, with projections indicating a quarter increase by 2030.^6^

To generate a profound knowledge about the dimensions of financial burdens and to identify patients at risk of experiencing financial distress, precise measurements are required. Since their inception within the United States health care system, definitions and measurement instruments have predominantly been tailored to United States-specific characteristics, such as the COST questionnaire by de Souza et al.^7^ The transferability to universal health care systems is hindered, however, due to fundamental differences of the systems. Beyond the general considerations involved in adapting United States-specific instruments to other countries, measurement instruments need to address the multidimensionality and complexity of the financial challenges or allow for a more detailed and focused analysis of relevant dimensions such as changes in the financial situation, subjective financial distress or its impact on the QoL.

Some country-specific approaches for universal health care systems do account for the country-specific health care regulations and aspects. The Italian 18-item Patient Reported Outcome For Fighting Financial Toxicity (PROFFIT) by Riva et al.^8^ is the first instrument developed in a country with a fully public health care system and focuses on financial burden and its determinants. Its direct applicability to Germany is limited as the health care systems and financial landscape differ. For example, Germany’s mandatory health insurance significantly lowers individual health care costs, limiting out-of-pocket payments (OOPPs). Administrative regulations are more complex, however, and navigating the health care and insurance systems during illness can be complicated and distressing for patients. In contrast, Italy’s national health service varies regionally, and although it offers universal coverage, gaps in coverage or longer waiting times may result in higher OOPPs. Another important reason for developing a new instrument for Germany and similar contexts is that existing instruments often focus on OOPPs and neglect potential financial distress caused by reduced labor activity and the resulting income loss. So, the nine-item Financial Index of Toxicity (FIT), developed by Hueniken et al.^9^ in 2020, primarily focuses on assessing the objective financial burden and lost productivity resulting from cancer treatment in Canada. It does not comprehensively account for subjective financial distress, however, which can be particularly relevant in the context of changing employment and income situations. Thus, the aim of the questionnaire development was to focus on the subjective financial distress due to the cancer diagnosis, while considering the specific circumstances of patients and the health care system in the German context.

## Background: Conceptualization of Financial Burden due to cancer

Financial burden is understood as a multidimensional concept including objective financial burden, consisting of direct medical costs (e.g. for medications, therapies, or medical aids), direct non-medical costs (e.g. household, leisure, or work-related costs), as well as indirect costs, especially loss of income. Moreover, it includes subjectively perceived distress, that is currently conceptualized through three domains: (i) material conditions, (ii) psychosocial responses, and (iii) behavioral coping.^1^^,^^10^ To describe financial burden associated with cancer, the term ‘financial toxicity’ has been established in the USA, reflecting the extent to which something is harmful to cancer patients. We propose, however, to recognize the financial challenges as the overall ‘financial effects of cancer experienced by patients’ instead of describing it in terms of ‘financial toxicity’ to emphasize the multi-dimensionality of the phenomenon rather than focusing on the outcome itself.

**Figure 1:**
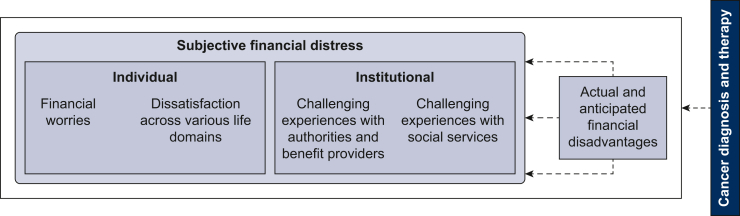
Conceptual model to describe financial effects of cancer experienced by patients in Germany.

During preliminary studies (see Supplementary material S1, available at https://doi.org/10.1016/j.esmoop.2024.103992 for main project steps) we developed a conceptual model for financial burden of German cancer patients (Figure 1). Identification of primary dimensions as well as item generation was guided by established frameworks of Financial Toxicity^1^^,^^10^^,^^11^ and a systematic literature review (*n* = 46) carried out by us.^12^ We also conducted semi-structured interviews with cancer patients (*n* = 18), using an interview guideline developed based on the relevant literature.^1^^,^^10^^,^^11^ Furthermore, we conducted focus groups with representatives of social services (*n* = 4) from outpatient cancer counselling and acute hospitals in Germany. The transcripts underwent qualitative content analysis, leading to major dimensions and topics contributing to financial distress.^13^

Subjective financial distress can be experienced on an individual and institutional level. On the individual level, it is perceived as financial worries and dissatisfaction across various life domains and on the institutional level as challenging experiences with authorities and benefit providers. [The initial instrument also comprised the domain of challenging experiences with social services. Due to insufficient test-retest reliability, it is not included in the final patient-reported outcome measure (PROM) (see Supplementary material S2, available at https://doi.org/10.1016/j.esmoop.2024.103992). Furthermore, patients may apply different cognitive and behavioral strategies, which would be less and more effective in coping with the burdensome situation. The present measurement instrument focusses on subjective financial distress resulting from cancer and does not include coping strategies, which were evaluated and are going to be published elsewhere.]

Financial worries encompass concerns related to financial life with cancer or ensuring financial security for oneself and one’s family. They reflect the patient’s emotional response to the potentially changed financial situation, including the cognitive evaluation of (anticipated) financial disadvantages. The other component of financial distress on an individual level are perceived financial restrictions in various life domains, including lifestyle, holidays, leisure time, living situation, and relationships with friends and family. This includes domain-related financial satisfaction and experienced burden resulting from the corresponding financial restrictions. On the institutional level, experiences with authorities and benefit providers address challenges such as feeling pressured to make quick decisions, lacking relevant information about the financial support and the administration process or fearing financial disadvantages. Subjective financial distress is dependent on (expected) changes of the objective financial situation and financial loss resulting from the disease and therapy. It comprises direct medical and non-medical as well as indirect costs (e.g. loss of income).

The domains of subjective financial distress illustrate their dependence on the German health care system. Besides systemic factors, cultural aspects also shape the evaluation of relevant aspects of financial distress and the identified personal worries due to socialization.

## Methods

The PROM was newly developed; development and validation adhered to the RatSWD quality standards for measurement instruments^14^ and conforms with the Standards for Educational and Psychological Testing developed by the American Educational Research Association, American Psychological Association, and National Council on Measurement in Education.^15^ See Supplementary material S1, available at https://doi.org/10.1016/j.esmoop.2024.103992 for an overall flow chart showing the major steps for instrument development and validation. This study represents the steps from the quantitative evaluation of the questionnaire up to the final confirmation of scales and items.

### Financial Distress of Cancer Assessment Tool

See Table 1 for the finalized and validated subscales and items constituting the PROM. See Supplementary material S3, available at https://doi.org/10.1016/j.esmoop.2024.103992 for the assessment of income changes and OOPPs, which were not subjects of validation but can be used by researchers to assess actual and anticipated financial disadvantages. See Supplementary material S4, available at https://doi.org/10.1016/j.esmoop.2024.103992 for the scoring procedure.

**Table 1:** Final Financial Distress of Cancer Assessment Tool (FIAT)

No.	Label	German	English (for comprehension only)	Orientation	Subscale
		**Worüber machen Sie sich Sorgen? Inwieweit trifft eine Aussage zu?**	**What are your concerns? To what extent does a statement apply?**		
1	Life	Ich mache mir Sorgen darüber, wie ich mit meiner Krebserkrankung mein Leben finanzieren soll.	I worry about how to finance my life with my cancer diagnosis.	+	Financial worries
2	Expenses	Ich mache mir Sorgen darüber, wie ich die Ausgaben aufgrund der Krebserkrankung decken kann.	I worry about how I can cover the expenses due to the cancer diagnosis.	+	Financial worries
3	Family	Ich mache mir Sorgen um die finanzielle Absicherung meiner Angehörigen (Kinder oder andere Familienmitglieder).	I worry about the financial security of my dependents (children or other family members).	+	Financial worries
4	Protect	Ich mache mir Sorgen, dass ich zu wenig Geld für meine Zukunft zurücklegen kann.	I worry that I won’t be able to save enough money for my future.	+	Financial worries
		**In den folgenden Fragen geht es um die Auswirkung Ihrer finanziellen Situation auf Ihre Zufriedenheit in verschiedenen Lebensbereichen.**	**In the following questions, we inquire about the impact of your financial situation on your satisfaction in various areas of life.**		
5	Lifestyle satisfaction	Wie zufrieden sind Sie mit Ihren finanziellen Möglichkeiten, eine gesunde Lebensweise zu realisieren?	How satisfied are you with your financial abilities to achieve a healthy lifestyle?	-	Impact on life
6	Lifestyle burden	Wie stark fühlen sie sich dadurch belastet?	How much do you feel burdened by this?	+	Impact on life
7	Vacation satisfaction	Wie zufrieden sind Sie mit Ihren finanziellen Möglichkeiten, Ihren Urlaub zu gestalten?	How satisfied are you with your financial abilities to plan your vacation?	−	Impact on life
8	Vacation burden	Wie stark fühlen sie sich dadurch belastet?	How much do you feel burdened by this?	+	Impact on life
9	Leisure time satisfaction	Wie zufrieden sind Sie mit Ihren finanziellen Möglichkeiten, Ihre Freizeit zu gestalten?	How satisfied are you with your financial abilities to plan your leisure time?	−	Impact on life
10	Leisure time burden	Wie stark fühlen sie sich dadurch belastet?	How much do you feel burdened by this?	+	Impact on life
11	Living satisfaction	Wie zufrieden sind Sie mit Ihren finanziellen Möglichkeiten zur Gestaltung Ihrer Wohnsituation?	How satisfied are you with your financial abilities to design your living situation?	−	Impact on life
12	Living burden	Wie stark fühlen sie sich dadurch belastet?	How much do you feel burdened by this?	+	Impact on life
13	Social satisfaction	Wie zufrieden sind Sie mit Ihren finanziellen Möglichkeiten, Ihre Beziehungen zu Freunden und Familie zu gestalten?	How satisfied are you with your financial abilities to shape your relationships with friends and family?	−	Impact on life
14	Social burden	Wie stark fühlen sie sich dadurch belastet?	How much do you feel burdened by this?	+	Impact on life
		**Wie erleben Sie den Umgang und die Kommunikation mit Behörden und Leistungsträgern (z.B. Krankenkasse, Rentenversicherung, Agentur für Arbeit)?**	**How do you experience dealing with and communicating with authorities and benefit providers (e.g. health insurance, pension insurance, employment agency)?**		
15	Pressure	Ich fühle mich unter Druck gesetzt, Entscheidungen schnell zu treffen.	I feel pressured to make decisions quickly.	+	Challenges Authorities
16	Accuracy	Ich habe Angst, dass ich Formulare oder Anträge falsch ausfülle.	I’m afraid I’ll fill out forms or applications incorrectly.	+	Challenges Authorities
17	Waiting times	Ich fühle mich durch lange Wartezeiten bei der Bearbeitung von Anträgen verunsichert.	I feel insecure due to long waiting times in the processing of applications.	+	Challenges Authorities
18	Disadvantages	Ich habe Angst, dass mir finanzielle Nachteile entstehen.	I’m afraid of facing financial disadvantages.	+	Challenges Authorities
19	Communication	Ich empfinde die Kommunikation zwischen verschiedenen Stellen als unzureichend.	I find the communication between different entities inadequate.	+	Challenges Authorities

Note. Response categories for items 1-4, 1 = trifft überhaupt nicht zu to 5 = trifft voll und ganz zu (English translation for comprehension only: 1 = does not apply at all to 5 = applies fully); response categories for items 5, 7, 9, 11, 13, 1 = überhaupt nicht zufrieden to 5 = sehr zufrieden (English translation for comprehension only: 1 = not satisfied at all to 5 = very satisfied; response categories for items 6, 8, 10, 12, 14, 1 = überhaupt nicht to 5 = sehr (English translation for comprehension only: 1 = not at all to 5 = very; response categories for items 15-19: 1 = trifft überhaupt nicht zu to 5 = trifft voll und ganz zu, -98 = hatte bisher keinen Kontakt mit den entsprechenden Stellen (English translation for comprehension only: 1 = does not apply at all to 5 = applies fully, -98 = had no contact with the corresponding institutions so far).

### Patients and recruitment

Participants were recruited at two different time points (T1: June to July 2022; and T2: April to August 2023) from the outpatient clinic and ambulances at the National Center for Tumor Diseases (NCT), Heidelberg, as well as the conservative day care unit and oncological ward B100 at the University Hospital Jena. Study nurses daily screened patients for eligibility. Patient information and consent forms were provided to eligible patients, who were then informed about the study by physicians. Signed consent forms were collected by study nurses, who distributed self-administered paper–pencil questionnaires on site.

### Eligibility criteria

Eligibility criteria comprised patients aged ≥18 years who were treated at the NCT Heidelberg and University Hospital Jena with all types of cancer who had undergone at least 2 months of cancer-related therapy with an Eastern Cooperative Oncology Group (ECOG) status <2, and had given informed consent to participate in the study.

### Statistical analysis

An initial examination of the measurement instrument was carried out with data from T1. Descriptive analysis, including distribution analyses (Shapiro–Wilk tests) and assessment of skew and excess with evidence for possible ceiling and floor effects, was carried out. Identified items with floor effects have been excluded from further analyses. Factorial validity was assessed via exploratory factor analysis (EFA). Preliminary reliability was assessed calculating the factor analysis-based composite general reliability coefficient Raykov’s rho. Using data of T2, the modified instrument was examined in its final form. Confirmatory factor analyses (CFA) were carried out on data of T2 to confirm the factorial structure of the PROM identified at T1. The model fit of CFAs was evaluated using the chi-square test (CMIN), the root mean square error of approximation (RMSEA), the comparative fit index (CFI), and Tucker–Lewis index (TLI).^16^ A CFI and TLI of ≥0.95, along with an RMSEA of 0.08 or lower indicate an acceptable fit.^17^

To assess construct validity Pearson correlations between the subjective financial distress measured by the Financial Distress of Cancer Assessment Tool (FIAT) scales and predefined concepts, measured by established instruments, were calculated. To support convergent validity, significant (*P* < 0.001) moderate to strong correlations of *r* = |0.3| or higher were expected. For divergent validity, correlations were expected to be non-significant (*P* > 0.05) with scores not higher than *r* = |0.3|. The criterion validity of the PROM was assessed through Pearson correlations and linear regression analysis between the FIAT scales and health outcomes possibly affected by financial distress. We expected significant (*P* < 0.001) correlations (regression coefficients) of ≥|0.3|.

Convergent validity was assessed by using the single item EORTC-QLQ-C30 financial distress subscale (Q28),^18^ financial worries [Worry Domains Questionnaire (WDQ^19^)], general distress [National Comprehensive Cancer Network (NCCN) distress thermometer^20^] and burden due to uncertainties of the Stress and Coping Inventory (SCI^21^). Divergent validity was assessed using a social desirability scale [Balanced Inventory of Desirable Responding (BIDR)^22^] and a personality (Big-5) inventory.^23^ Criterion validity was evaluated using items 29 and 30 of the EORTC-QLQ-C30 to measure health-related QoL (HRQoL)^18^ and the Short Form Patient Health Questionnaire (PHQ-4) to assess depression and anxiety.^24^ Furthermore, we assessed income changes using the 2016 Demographic Standards,^25^ which were adapted to the target population. The evaluation of OOPPs was drawn from theory on frameworks of financial toxicity^1^^,^^10^ as well as empirical studies assessing OOPPs of cancer patients in Germany.^26^, ^27^, ^28^ The items were discussed within the research team and tested in a cognitive pretest^29^ (see Supplementary material S3, available at https://doi.org/10.1016/j.esmoop.2024.103992).

CFA was carried out using Mplus 8 and EFA and all other analyses using IBM SPSS Statistics 29. Reliability was obtained by Raykov’s rho and test–retest reliability was measured using bivariate Pearson correlations between data collected at T1 and from a subset of patients at T2 who were surveyed at both time points, with sample sizes ranging from 51 to 61. Linear regression analysis was used to identify risk factors that have an effect on subjectively perceived financial distress measured by the FIAT scales. Significance levels were set at α = 0.05 for all analyses. Sample sizes between 100 and 300 participants were considered adequate for validation purposes in correlation-based analyses^30^ and for conducting CFA.^31^ See the study protocol^32^ for a comprehensive description of analyses applied.

## Results

### Patient characteristics

Table 2 presents the demographic and clinical characteristics of participants at T1 and T2. At T1, 111 patients completed the pre-final PROM, which underwent refinement before being redistributed at T2 to participants from T1 as well as additional participants. A total of 73 participants from T1 as well as 194 additional participants took part at T2, resulting in a combined sample size of 267 at T2. The majority of participants were female (T1: 56.8%, T2: 67.7%), with a mean age of 54.8 years (range 18-79 years) in T1 and 59.0 years (range 20-83 years) in T2. Most patients had statutory insurance (T1: 89.4%, T2: 83.5%). Gynecological cancer was the predominant cancer entity (T1: 31.5%, T2: 38.5%), followed by gastrointestinal tumors (T1: 26.1%, T2: 23.3%) and hematological malignant disease (T1: 23.4%, T2: 16.4%). Approximately half of the patients were included during initial treatment, including adjuvant and maintenance therapy. Chemotherapy was the predominant therapy (T1: 87.4%, T2: 83.1%), followed by surgery (T1: 48.6%, T2: 50.6%). When comparing the occupational status before the diagnosis and at the time of the survey there was a decrease in the percentage of patients working full- or part-time, while the percentage of patients who were non-employed increased. The overall response rate (RR6, AAPOR^33^) for T1 was 65%, while at T2, 67% of patients from T1 and 63% of newly approached patients participated.

**Table 2:** Samples and patient characteristics

Sample size	T1	T2
	111	267
Survey period	June-July 2022	March-August 2023
Survey Mode	Paper–pencil	Paper–pencil
Gender, *n* (%)		
Female	63 (56.8)	174 (67.7)
Male	48 (43.2)	83 (32.3)
Age, years, *n* (%)		
<40	10 (9.3)	12 (5.0)
40 to 49	24 (22.4)	45 (18.7)
50 to 59	21 (19.6)	70 (29.0)
60 to 75	51 (47.7)	106 (44.0)
>75	1 (0.9)	8 (3.3)
Insurance status, *n* (%)^a^		
Statutory health insurance	93 (89.4)	223 (90.3)
Private health insurance	10 (9.6)	24 (9.7)
Entitlement to assistance (i.e. civil servants, members of the police force)	1 (1.0)	15 (6.1)
Not insured	0 (0)	1 (0.4)
Educational attainment, *n* (%)		
Less than primary education	0 (0.0)	1 (0.4)
Lower secondary education	6 (6.0)	20 (8.4)
Upper secondary education	46 (46.0)	112 (46.9)
Postsecondary education	48 (48.0)	106 (44.4)
Cancer entity, *n* (%)^b^		
Gastrointestinal tumor	29 (26.1)	60 (23.3)
Gynecological tumor	35 (31.5)	99 (38.5)
Head and neck tumor	4 (3.6)	6 (2.3)
Hematological malignant disease	26 (23.4)	42 (16.4)
Skin cancer	4 (3.6)	20 (7.8)
Lung cancer	4 (3.6)	11 (4.3)
Urogenital tumor	3 (2.7)	12 (4.7)
Other	15 (13.6)	39 (15.2)
Treatment phase, *n* (%)^c^		
Planning phase after diagnosis (therapy has not yet begun)	2 (2.2)	1 (0.4)
During initial treatment, including adjuvant therapy and maintenance therapy	48 (51.6)	117 (52.5)
Follow-up care	8 (8.6)	34 (15.2)
Treatment completed	4 (4.3)	5 (2.2)
Treatment of relapse: curative	11 (11.8)	24 (10.8)
Treatment of relapse: palliative	20 (21.5)	42 (18.8)
Treatment(s), *n* (%)^b^		
Immunotherapy	32 (28.8)	83 (31.1)
Surgery	54 (48.6)	135 (50.6)
Radiotherapy	28 (25.2)	80 (30.0)
Chemotherapy	97 (87.4)	222 (83.1)
(Anti-) hormone therapy	8 (7.)	31 (11.6)
Antibody therapy	32 (28.8)	86 (32.2)
Watch and wait	4 (3.6)	12 (4.5)
Complementary medicine	6 (5.4)	17 (6.4)
Other	8 (7.2)	10 (3.7)
Occupational status before diagnosis, *n* (%)		
Full-time	54 (51.9)	104 (40.5)
Part-time	21 (20.29	64 (24.0)
Alternative forms of employment^d^	6 (5.8)	15 (5.6)
Not employed	23 (22.1)	74 (28.8)
Occupational status after diagnosis, *n* (%)		
Full-time	41 (40.2)	51 (28.5)
Part-time	18 (17.6)	43 (24.0)
Alternative forms of employment	4 (3.9)	10 (5.6)
Not employed	39 (38.2)	75 (41.9)

Note. Deviations in *n* due to data missing.

a T1, presented as single-choice question; T2, presented as multiple-choice question.

b Presented as multiple-choice question.

c Categories T1: 1, after diagnosis (therapy has not yet begun); 2, during initial treatment, including adjuvant therapy and maintenance therapy; 3, initial treatment completed; 4, follow-up care; 5, treatment of relapse; 6, advanced treatment/palliative treatment; categories T 2: 1, after diagnosis (therapy has not yet begun); 2, during initial treatment, including adjuvant therapy and maintenance therapy; 3, follow-up care; 4, treatment completed; 5, treatment of relapse: curative; 6, treatment of relapse: palliative.

d Marginally employed, ‘One-Euro-Job’, occasional or irregularly employed, in vocational training/apprenticeship, in retraining, voluntary military service, Federal Voluntary Service or Voluntary Social Year, maternity leave, parental leave, or other forms of leave.

### Item generation and selection

**Figure 2:**
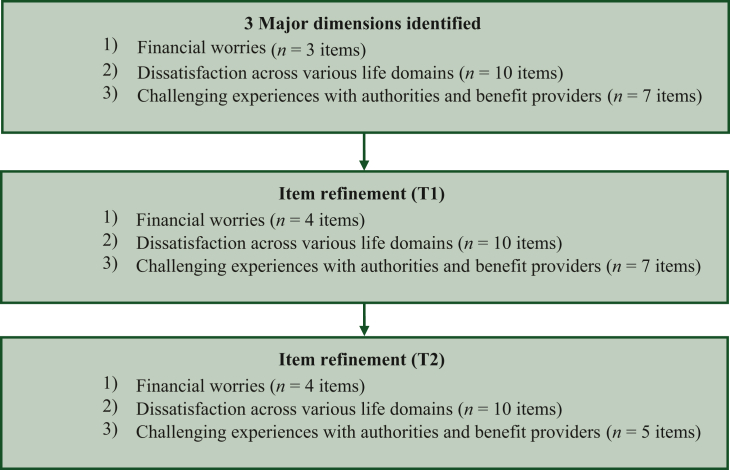
Flow chart of item selection.

Item generation was based on the results of the qualitative interviews with patients^13^ and representatives of social services. In a cognitive pretest^29^ (*n* = 16) formulated items were refined and tested in two quantitative surveys (*N1* = 111; *N2* = 267). In CFA, items with factor loadings >|0.3| were omitted from the final instrument. Additionally, items demonstrating a test–retest reliability >0.3 were also excluded from the instrument. See Figure 2 for the flow chart of item selection. See Supplementary material S2, available at https://doi.org/10.1016/j.esmoop.2024.103992 for items excluded during item selection.

### Study population

#### Confirmatory factor analysis

Financial worries and dissatisfaction across various life domains were modelled as two factors of individual financial distress which was supported by the results: CMIN (χ^2^) = 889.303 (df = 36), *P* < 0.001; RMSEA = 0.078, *P* < 0.05; CFI = 0.95; TLI = 0.93. For financial worries standardized factor loadings ranged from 0.71 (protect) to 0.96 (life). For the dissatisfaction with various life domains, loadings ranged from 0.55 (lifestyle) to 0.76 (social). The correlation between the two factors was 0.65 (*P* < 0.001) (see Supplementary material S5, available at https://doi.org/10.1016/j.esmoop.2024.103992).

Challenging experiences with authorities and benefit providers as well as challenging experiences with counselling services were modelled as two factors of institutional financial distress. This factor structure was supported in the data: CMIN (χ^2^) = 649.406 (df = 36), *P* < 0.001; RMSEA = 0.082, *P* < 0.05; CFI = 0.93; TLI = 0.91. The standardized factor loadings of the first factor ranged from 0.62 (pressure) to 0.91 (waiting time) and for the second factor from 0.57 to 0.85 (see Supplementary material S6, available at https://doi.org/10.1016/j.esmoop.2024.103992). The correlation between the two factors was 0.20 (*P* < 0.001). See Supplementary material S7, available at https://doi.org/10.1016/j.esmoop.2024.103992 for item statistics.

#### Reliability

A good to excellent composite reliability,^34^ as indicated by Raykov’s rho was obtained ranging from 0.88 for challenging experiences with authorities to 0.96 for dissatisfaction across various life domains. Furthermore, the test–retest analysis, revealed correlations ranging from 0.64 (*P* < 0.001) for challenging experiences with authorities to 0.75 (*P* < 0.001) for financial concerns (see Supplementary material S8, available at https://doi.org/10.1016/j.esmoop.2024.103992). Notably the scale for dissatisfaction with counselling services was excluded from further analyses as the test–retest reliability was found to be low (<0.3).

#### Construct validity

We observed moderate to high correlations between the FIAT scales and the related concepts of the EORTC-QLQ-C30 financial distress subscale^18^ ranging from 0.47 to 0.61 (*P* < 0.001), financial worries (WDQ^19^) ranging from 0.59 to 0.85 (*P* < 0.001), general distress (NCCN distress thermometer^20^) ranging from 0.37 to 0.47 (*P* < 0.001), and burden due to uncertainties (SCI^21^) ranging from 0.53 to 0.63 (*P* < 0.001). Additionally, we identified low correlations between the FIAT scales and concepts assumed to be unrelated, namely social desirability (BIDR^22^) (ranging from 0.02 to 0.1, *P* > 0.05), and personality (Big-5 inventory^23^) (ranging from 0.00 to 0.21, *P* > 0.05 to *P* < 0.05) (for a detailed overview, see Supplementary material S9 and S10, available at https://doi.org/10.1016/j.esmoop.2024.103992). Thus, construct validity could be supported.

#### Criterion validity

Significant correlations have been observed between HRQoL and FIAT scales, ranging from −0.21 to −0.32 (*P* < 0.001), indicating a negative effect of financial distress on HRQoL. Correlations for depression ranged from 0.35 to 0.46 (*P* < 0.001), suggesting higher depression scores with increased financial distress. Similarly, correlations for anxiety ranged from 0.27 to 0.40 (*P* < 0.001), indicating higher anxiety scores with increased financial distress (see Supplementary material S11, available at https://doi.org/10.1016/j.esmoop.2024.103992 for a comprehensive overview). Additionally, regression analysis showed a significant effect (*P* < 0.05) of income reduction and subjective socioeconomic status (SSS) measured by subjective class identification^35^ on the financial worries subscale of the PROM, indicating that income reduction as well as a low SSS is significantly associated with high scores of financial worries. In contrast, major patient characteristics (e.g. age, gender, living alone versus living with a partner) did not have a significant effect on financial worries (see Supplementary material S12, available at https://doi.org/10.1016/j.esmoop.2024.103992). Thus, criterion validity could be supported.

## Discussion

The final 19-item PROM consists of three subdomains, each capturing different aspects of subjective financial distress experienced by cancer patients: (i) financial worries; (ii) dissatisfaction across various life domains, and (iii) challenging experiences with authorities and benefit providers (e.g. employment agency, health insurance).

All subdomains demonstrated strong psychometric properties, including high reliability and validity, as evidenced by CFA, correlational, and linear regression analyses. Composite reliability was rated as good to excellent for all three subscales. Notably, it was particularly high for dissatisfaction across various life domains and financial worries. Furthermore, all three subscales exhibited sufficient to high test–retest reliability. Concerning convergent construct validity, moderate to high correlations between the instrument’s subscales and established measures of financial distress (Q28 of EORTC-QLQ-C30^18^), financial worries (WDQ^19^), general distress (NCCN distress thermometer^20^), and burden due to uncertainties (SCI^21^), support its convergent construct validity. The three FIAT scales particularly closely relate to ‘financial worries’ as measured with the WDQ,^19^ but enrich this concept by additional focus on worries resulting from the cancer disease, the impact on different life areas, and distress resulting from interactions with authorities and benefit providers.

A further notable strength of the PROM lies in its multidimensional approach as it considers not only economic concerns, but also their impact on various aspects of patients’ lives and interactions with institutions. This approach differs from the single-item measurement of financial effects over the past week within the EORTC-QLQ-C30 (Q28),^18^ which has been often applied in previous studies. It overlooks the multi-dimensionality and concentrates on a short time period rather than a holistic contextualization. Our analysis, however, demonstrated a moderate to high correlation between the FIAT scales and Q28 of the EORTC-QLQ-C30.^18^ Thus, similar understandings of the concept are measured by both approaches, but the intentions of the instruments differ. Our analysis also showed a significant correlation between FIAT domains and HRQoL (Q29 and Q30 of the EORTC QLQ-C30).^18^ Given that financial distress is a crucial mediator for HRQoL and greatly affects cancer patients, the FIAT instrument could prove to be beneficial in capturing the multidimensionality of this construct.

Comparing the PROM with previous instruments implemented in the United States context like the COST measurement,^7^ it reflects the fundamental differences between the systems. Most of the 11 COST items focus on the impact of additional medical expenses and access to treatment due to personal financial distress, which does not seem to be appropriate for the German health care context. As the COST instrument also covers financial worries, however, it supports our understanding of universal domains within the instrument, even though we expanded it further by accounting for universal health care characteristics in terms of ‘challenging experiences with authorities and benefit providers’. This is supported by the Italian PROFFIT questionnaire that addresses the Italian universal health care system,^8^ incorporating the systemic level. We argue, however, to incorporate these challenging experiences as part of the construct of financial distress rather than as a determinant, as our patient interviews showed that navigation through the system can be stressful in itself.^13^ Similar to our approach, PROFFIT focuses on assessing subjective financial distress. Arenare et al.^36^ examined correlations between the PFOFFIT score and Q28 of the EORTC-QLQ-C30. We obtained correlations of comparable size and direction showing a similar capacity for both instruments in capturing financial burden among cancer patients, while also accounting for the specific country characteristics in their assessment.

In contrast, Canada’s 9-item FIT^9^ primarily focuses on assessing the objective financial burden and lost productivity resulting from cancer treatment, without directly addressing subjective financial distress. We suggest, however, to assess impacts of financial distress on different areas of living as in our instrument rather than assessing the overall degree of financial burden and lost productivity to better understand in which life areas the patients are confronted with financial difficulties. It could aid in guiding the implementation of counseling services and supportive interventions. The FIT demonstrated low positive correlations of financial toxicity with income loss due to cancer. Similarly, for the financial worries subscale in our analysis a low significant effect of income loss was found. To be able to connect the financial distress to the actual (worsened) financial situation, we suggest researchers use the questions we used to assess the change in income and out-of-pocket costs (see Supplementary material S3, available at https://doi.org/10.1016/j.esmoop.2024.103992).

While there is a need for tailored and country-specific solutions, the domains identified could be transferred to other third party paid health care systems. The questions themselves, however, should be revised and culturally adapted.

Some limitations of the study need to be acknowledged. The initial domains of the instrument also covered behavioral and cognitive coping strategies to mitigate financial distress but were eliminated from the final instrument due to insufficient psychometric results. Partially validated scales measuring coping strategies will be published elsewhere. Since our prestudies demonstrated the importance of coping strategies as the part of the construct of financial effects, further studies should investigate them in more depth. The developed instrument was administered to patients during their regular doctor visits at the two participating hospitals, resulting in a convenience sample rather than a probability sample. Therefore, the generalizability of the findings may be limited. Furthermore, although we included patients from different treatment stages, the instrument has not been tested in cancer survivors yet. The suggested scoring simplifies the evaluation of financial effects by calculating the mean scores for items/domains, that might not be subjectively equally important to the patients, even though we incorporated the opinion and their values within our prestudies.

Furthermore, the presented study focused mainly on a cross-sectional perspective to test the validity of the instrument. As financial distress elaborates and changes over time, however, future studies could explore the sensitivity of the instruments to these changes to time. This could support longitudinal studies in the future, which are necessary to monitor patients’ financial distress of a cancer disease over a period of time to understand the phenomenon in more depth. Additionally, other variables, such as the time of diagnosis and changes in employment status, should also be investigated to provide a more comprehensive understanding of financial distress in cancer patients. As the instrument was developed to be applicable in social service counseling and for psychosocial support, its utility in informing interventions aimed at alleviating the financial impact of cancer on patients’ well-being and QoL should be further investigated. Additionally, the insights gained from applying the FIAT instrument could inform the development of guidelines to better assist patients in managing the financial challenges associated with their condition.

### Conclusions

Financial distress is a common phenomenon of a cancer disease, which needs to be measured precisely. While Germany’s health care system, with its comprehensive health insurance coverage and social welfare provisions, helps reduce cancer-related medical costs, the subjective financial distress experienced by patients can be elevated by the complexities of navigating the system. Individual experiences of financial distress may be transferable to other health care systems, whereas institutional factors are country-specific and require tailored considerations.

The FIAT represents a reliable and valid tool for identifying and addressing financial distress among cancer patients in Germany that can be used in research and clinical practice. It might also be suitable for social service counseling, psycho-oncological support, as well as its integration as a patient-reported outcome in clinical studies which should be tested in further studies.

## References

[1] Lueckmann S.L., Kowalski C., Schumann N. (2021). Finanzielle Toxizität einer Krebserkrankung [Financial toxicity of cancer]. Onkologe.

[2] Longo C.J., Fitch M., Deber R.B., Williams A.P. (2006). Financial and family burden associated with cancer treatment in Ontario, Canada. Support Care Cancer.

[3] Mehlis K., Witte J., Surmann B. (2020). The patient-level effect of the cost of Cancer care - financial burden in German Cancer patients. BMC Cancer.

[4] Ramsey S.D., Bansal A., Fedorenko C.R. (2016). Financial insolvency as a risk factor for early mortality among patients with cancer. J Clin Oncol.

[5] Longo C.J., Fitch M.I., Banfield L., Hanly P., Yabroff K.R., Sharp L. (2020). Financial toxicity associated with a cancer diagnosis in publicly funded healthcare countries: a systematic review. Support Care Cancer.

[6] Zentrum für Krebsregisterdaten (2021). Gesellschaft der epidemologischen Krebsregister in Deutschland E.V. Krebs in Deutschland für 2017/2018. Robert Koch Institut. https://www.krebsdaten.de/Krebs/DE/Content/Publikationen/Krebs_in_Deutschland/kid_2021/krebs_in_deutschland_2021.pdf?__blob=publicationFile.

[7] de Souza J.A., Yap B.J., Hlubocky F.J. (2014). The development of a financial toxicity patient-reported outcome in cancer: the COST measure. Cancer.

[8] Riva S., Arenare L., Di Maio M. (2021). Cross-sectional study to develop and describe psychometric characteristics of a patient-reported instrument (PROFFIT) for measuring financial toxicity of cancer within a public healthcare system. BMJ Open.

[9] Hueniken K., Douglas C.M., Jethwa A.R. (2020). Measuring financial toxicity incurred after treatment of head and neck cancer: development and validation of the Financial Index of Toxicity questionnaire. Cancer.

[10] Witte J., Mehlis K., Surmann B. (2019). Methods for measuring financial toxicity after cancer diagnosis and treatment: a systematic review and its implications. Ann Oncol.

[11] Carrera P.M., Kantarjian H.M., Blinder V.S. (2018). The financial burden and distress of patients with cancer: understanding and stepping-up action on the financial toxicity of cancer treatment. CA Cancer J Clin.

[12] Pauge S., Surmann B., Mehlis K. (2021). Patient-reported financial distress in cancer: a systematic review of risk factors in universal healthcare systems. Cancers.

[13] Züger A, Mathies V, Mehlis K, et al. Self-Reported Determinants for Subjective Financial Distress: A Qualitative Interview Study with German Cancer Patients. Preprint 2023. 10.1136/bmjopen-2023-081432 PMC11781118 39880449

[14] Quality Standards Working Group. Quality Standards for the Development, Application, and Evaluation of Measurement Instruments in Social Science Survey Research. RATSWD Working Paper 245; 2015.

[15] American Educational Research Association (2014). Standards for Educational and Psychological Testing.

[16] Beauducel A., Wittmann W.W. (2005). Simulation study on fit indexes in CFA based on data with slightly distorted simple structure. Struct Equ Modeling.

[17] Hu L., Bentler P.M. (1999). Cutoff criteria for fit indexes in covariance structure analysis: conventional criteria versus new alternatives. Struct Equ Modeling.

[18] European Organisation for Research and Treatment of Cancer EORTC QLQ-C30 (Version 3.0). http://racctrial.org/wp-content/uploads/2019/09/QLQ-C30-German.pdf.

[19] Stöber J. (1995). Besorgnis: Ein Vergleich dreier Inventare zur Erfassung allgemeiner Sorgen. [Worry: a comparison of three inventories for assessing general worry]. Zeitschrift für Differentielle und Diagnostische Psychologie.

[20] Mehnert A., Müller D., Lehmann C., Koch U. (2006). Die deutsche Version des NCCN Distress-Thermometers [The German version of the NCCN distress thermometer]. Zeitschrift für Psychiatrie, Psychologie und Psychotherapie.

[21] Satow L. (2012). Leibniz-Institut für Psychologie (ZPID) (Hrsg.), Open Test Archive.

[22] Winkler N., Kroh M., Spiess M. (2006).

[23] Rammstedt B., Kemper C.J., Klein M.C., Beierlein C., Kovaleva A. (2014). Big Five Inventory (BFI-10).

[24] Löwe B., Wahl I., Rose M. (2010). A 4-item measure of depression and anxiety: validation and standardization of the Patient Health Questionnaire-4 (PHQ-4) in the general population. J Affect Disord.

[25] Beckmann K., Glemser A., Heckel C. (2016). Demographische Standards. eine gemeinsame Empfehlung des ADM, Arbeitskreis Deutscher Markt- und Sozialforschungsinstitute e.V., der Arbeitsgemeinschaft Sozialwissenschaftlicher Institute e.V. (ASI) und des Statistischen Bundesamtes. 6th ed. Wiesbaden. https://www.statistischebibliothek.de/mir/servlets/MCRFileNodeServlet/DEMonografie_derivate_00001549%20Band17_DemographischeStandards1030817169004.pdf.

[26] Lueckmann S.L., Schumann N., Hoffmann L. (2020). ‘It was a big monetary cut’– a qualitative study on financial toxicity analysing patients’ experiences with cancer costs in Germany. Health Soc Care Community.

[27] Schröder S.L., Schumann N., Fink A., Richter M. (2020). Coping mechanisms for financial toxicity: a qualitative study of cancer patients' experiences in Germany. Support Care Cancer.

[28] Witte J., Surmann B., Batram M., Mehlis K., Winkler E., Greiner W. (2019). Krankheitskosten – Finanzielle Belastung von Krebspatienten – Evidenz für den deutschen Versorgungskontext [Medical expenses - financial burden on cancer patients - evidence for the German healthcare context]. Gesundh Ökon Qual Manag.

[29] Lenzner T., Schick L., Hadler P. (2022). Projekt “Finanzielle Auswirkungen einer Tumorer- krankung (FIAT)“.

[30] Cohen J. (1988).

[31] Wolf E.J., Harrington K.M., Clark S.L., Miller M.W. (2013). Sample size requirements for structural equation models: an evaluation of power, bias, and solution propriety. Educ Psychol Meas.

[32] Richter L., Pauge S., Mehlis K. (2022).

[33] AAPOR. Standard Definitions (2023). Final Dispositions of Case Codes and Outcome Rates for Surveys. https://aapor.org/wp-content/uploads/2023/05/Standards-Definitions-10th-edition.pdf.

[34] Bühner M. (2010). Munich.

[35] Kluegel J.R., Singleton R., Starnes C.E. (1977). Subjective class identification: a multiple indicator approach. Am Soc Rev.

[36] Arenare L., Porta C., Barberio D. (2023). Confirmatory validation analysis of the PROFFIT questionnaire to assess financial toxicity in cancer patients. ESMO Open.

