# Supplementary material for: Measuring financial distress in German cancer patients: development and validation of the Financial Distress of Cancer Assessment Tool (FIAT)
Source: ESMO Open. 2024 Nov 29;9(12):103992. doi: 10.1016/j.esmoop.2024.103992 (PMC11648195; doi:10.1016/j.esmoop.2024.103992)
Supplement: Supplementary material [file mmc1.docx]

**S1: Development and validation process of the Financial Distress of Cancer Assessment Tool (FIAT)**

**Systematic Literature Review** (*N* = 46)

**Qualitative Patient Interviews** (*N* = 18)

**Cognitive Pretest** (*N* = 16)

**T1: Quantitative Paper-Pencil Survey** (*N* = 111)

**T2: Quantitative Paper-Pencil Survey** (*N* = 267)

**Final Confirmation of Scales and Items**

**Development Phase**

**Evaluation Phase**

**2 Expert Focus Groups** (*N* = 6)

Note. Inclusion criteria for patients surveyed in qualitative patient interviews, the cognitive pretest, and quantitative paper-pencil surveys: Patients aged ≥18 years who were treated at the NCT Heidelberg and University Hospital Jena, with any type of cancer, who had undergone at least two months of cancer-related therapy, had an ECOG status <2, and had given informed consent to participate in the study. Patient recruitment: NCT Heidelberg: Day care unit and outpatient clinics. Jena: Conservative day care unit and oncological ward B100; Study nurses daily reviewed the patient scheduling board to identify eligible patients.

**S2: Items excluded during item selection**

| **No.** | **German** | **English translation (for comprehension only)** | **Polarity** | **Subscale** |
| --- | --- | --- | --- | --- |
|  | **Wie erleben Sie den Umgang und die Kommunikation mit Behörden und Leistungsträgern (z.B. Krankenkasse, Rentenversicherung, Agentur für Arbeit)?** | **How do you experience dealing with and communicating with authorities and benefit providers (e.g., health insurance, pension insurance, employment agency)?** |  |  |
| 1 | Ich fühle mich schlecht beraten. | I feel poorly advised. | + | Challenges Authorities |
| 2 | Ich fühle mich missverstanden. | I feel misunderstood. | + | Challenges Authorities |
|  | Wie erleben Sie den Umgang und die Kommunikation mit Beratungsstellen (z.B. Sozialdienst im Krankenhaus oder Rehaklinik, Krebsberatungsstelle)? |  |  |  |
| 3 | Ich fühle mich schlecht beraten. | I feel poorly advised. | + | Challenges Social Services |
| 4 | Ich fühle mich missverstanden. | I feel misunderstood. | + | Challenges Social Services |
| 5 | Ich fühle mich unter Druck gesetzt, Entscheidungen schnell zu treffen (z.B. Wiedereinstieg Arbeit, Erwerbsminderungsrente, Kuraufenthalt). | I feel pressured to make decisions quickly (e.g., returning to work, disability pension, spa stay). | + | Challenges Social Services |
| 6 | Ich fühle mich durch lange Wartezeiten verunsichert. | I feel uncertain due to long waiting times. | + | Challenges Social Services |
| 7 | Ich empfinde die Kommunikation zwischen verschiedenen Stellen als unzureichend (z.B. Kommunikation zwischen Sozialdienst und meinem Behandlungsteam). | I perceive the communication between different parties as inadequate (e.g., communication between social services and my treatment team). | + | Challenges Social Services |

*Note. Items 1 and 2 were excluded post CFA due to correlated error terms. Items 3 to 7 were excluded due to insufficient test-retest reliability (r<0.03).*

**Response specifications**

| **Items** | **Response categories** | **English translation (for comprehension only)** |
| --- | --- | --- |
| 1 - 7 | 1 = trifft überhaupt nicht zu  2 = trifft wenig zu  3 = trifft mittelmäßig zu  4 = trifft ziemlich zu  5 = trifft voll und ganz zu | 1 = doesn´t apply at all  2 = applies little  3 = applies partly  4 = applies quite a bit  5 = applies completely |

**S3: Assessment of Income changes and out-of-pocket-payments (OOPPs) at T2**

| **Item number** | **Items (German)** | **Items English (for comprehension only)** |
| --- | --- | --- |
| ***Income changes*** |  |  |
| **1** | Wie hoch war Ihr persönliches monatliches Nettoeinkommen vor Ihrer Krebsdiagnose insgesamt? Zum persönlichen Nettoeinkommen gehören die Lohneinkommen und Kapitaleinkommen (z.B. Einkommen aus Vermietung und Wertpapieren) nach Abzug von Steuern und Sozialabgaben. Darüber hinaus zählen zum persönlichen Nettoeinkommen ebenfalls regelmäßige Zahlungen wie Renten, Wohngeld, Kindergeld, BAföG, Unterhaltszahlungen. Setzen Sie bitte ein Kreuz. | What was your total monthly net personal income before your cancer diagnosis? Net personal income includes wage income and capital income (e.g. income from renting and securities) after deduction of taxes and social security contributions. In addition, personal net income also includes regular payments such as pensions, housing benefit, child benefit, BAföG, maintenance payments. Please put a cross in the box. |
| **2** | Hat sich Ihr persönliches Nettoeinkommen seit Ihrer Krebsdiagnose verringert, erhöht oder ist es in etwa gleichgeblieben? | Has your personal net income decreased, increased or remained more or less the same since your cancer diagnosis? |
| **3** | Wie hoch ist aktuell Ihr persönliches Nettoeinkommen insgesamt? | What is your current total personal net income? |
| ***Expenses for living and lifestyle*** | **Wie haben sich Ihre Ausgaben für Lebensunterhalt und Lebensführung seit Ihrer Diagnose verändert? Bei Veränderung geben Sie bitte auch Ihre Ausgaben in den letzten sechs Monaten an. Meine Ausgaben in folgenden Bereichen sind…/haben sich…** | **How have your living expenses and lifestyle changed since your diagnosis? If there has been a change, please specify your expenses in the last six months in the following areas... / have changed...** |
| **4** | Wohnen (z.B. Miete, Heizung, Strom) | Housing (e.g., rent, heating, electricity) |
| **5** | Lebensmittel | Food |
| **6** | Freizeitkosten (z.B. Freizeitkosten während stationärer Rehamaßnahmen, Fitnesskurse oder Kosten für Hobbies) | Leisure costs (e.g., leisure costs during inpatient rehabilitation measures, fitness classes, or hobby expenses) |
| **7** | Kleidung (z.B. aufgrund von Gewichtszu- oder -abnahme) | Clothing (e.g., due to weight gain or loss) |
| **8** | Pflegeprodukte (z.B. Shampoo, Körpercreme, Mundspüllösung) | Personal care products (e.g., shampoo, body cream, mouthwash) |
| **9** | Fahrt- und Parkkosten (z.B. Fahrten zur Apotheke, ambulanten Versorgung/ Untersuchungen, Krankenhaus) | Travel and parking costs (e.g., trips to the pharmacy, outpatient care/appointments, hospital) |

**S3 (continued): Assessment of Income changes and out-of-pocket-payments (OOPs) at T2**

| ***Expenses for therapies and services*** | **Welche Ausgaben für Therapien und Dienstleistungen sind außerdem dazu gekommen? Geben Sie bitte den Gesamtbetrag für die letzten sechs Monate in etwa an.** | **What expenses for therapies and services have been added? Please provide the total amount for the last six months approximately.** |
| --- | --- | --- |
| **10** | Stationäre und ambulante Krankenhausaufenthalte (z.B. Eigenbeteiligung, Einzelzimmerzuschlag oder Fernsehgebühren) | Inpatient and outpatient hospital stays (e.g., personal contribution, single room surcharge, or television fees) |
| **11** | Medikamente (z.B. Zuzahlungen zu verschreibungspflichtigen Medikamenten, rezeptfreie Medikamente) | Medications (e.g., co-payments for prescription medications, over-the-counter medications) |
| **12** | Zuzahlung zu verordneten begleitenden Therapien (z.B. Physiotherapie, Ergotherapie, Rehamaßnahmen) | Co-payment for prescribed accompanying therapies (e.g., physiotherapy, occupational therapy, rehabilitation measures) |
| **13** | Nahrungsergänzungsmittel und alternative Therapien, die von keinem Leistungsträger übernommen werden (Komplementärmedizinische Ansätze) | Dietary supplements and alternative therapies not covered by any service provider (complementary medical approaches) |
| ***Further Expenses*** | **Hatten Sie weitere, bisher nicht aufgeführte Ausgaben aufgrund Ihrer Krebserkrankung? Falls ja, geben Sie bitte die Art der Ausgaben und den jeweiligen Gesamtbetrag seit Ihrer Krebsdiagnose an.** | **Have you had other, previously unmentioned expenses due to your cancer diagnosis? If yes, please specify the type of expenses and the respective total amount since your cancer diagnosis.** |
| **14** | Art der Ausgaben 1 | Type of expense 1 |
| **15** | Art der Ausgaben 2 | Type of expense 2 |
| **16** | Art der Ausgaben 3 | Type of expense 3 |

**Response specifications**

| **Items** | **Response categories** | **English translation (for comprehension only)** |
| --- | --- | --- |
| 1, 3 | 1 = unter 1000€  2 = 1000€ bis unter 1.500€  3 = 1.500 bis unter 2.000€  4 = 2.000 bis unter 2.500€  5 = 2.500 bis unter 3.000€  6 = 3.000€ bis unter 3.500€  7 = 3.500€ bis unter 4.000€  8 = 4.000€ bis unter 4.500€  9 = 4.500 bis unter 5.000 €  10 = 5.000€ und höher  -99 = keine Antwort | 1 = under 1000€  2 = 1000€ to under 1,500€  3 = 1,500€ to under 2,000€  4 = 2,000€ to under 2,500€  5 = 2,500€ to under 3,000€  6 = 3,000€ to under 3,500€  7 = 3,500€ to under 4,000€  8 = 4,000€ to under 4,500€  9 = 4,500€ to under 5,000€  10 = 5,000€ and higher  -99 = no answer |
| 2 | 1 = Mein persönliches Nettoeinkommen hat sich verringert.  2 = Mein persönliches Nettoeinkommen hat sich erhöht.  3 = Mein persönliches Nettoeinkommen ist in etwa gleichgeblieben.  -99 = keine Antwort | 1 = My personal net income has decreased.  2 = My personal net income has increased.  3 = My personal net income has remained roughly the same.  -99 = no answer |
| 4-9 | 1 = gleich geblieben  2 = erhöht, Gesamtbetrag für die letzten 6 Monate in etwa (€)  3 = verringert, Gesamtbetrag für die letzten 6 Monate in etwa (€)  -99 = keine Antwort | 1 = remained the same.  2 = increased, total amount for the last 6 months approximately (€)  3 = decreased, total amount for the last 6 months approximately (€)  -99 = no answer |
| 10-13 | 1 = nein  2 = ja, Gesamtbetrag für die letzten sechs Monate in etwa  -99 = keine Antwort | 1 = no  2 = yes, total amount for the last six months approximately (€)  -99 = no answer |
| 14-16 | 1 = Ich hatte keine weiteren Ausgaben  2 = Ich hatte weitere Ausgaben, bitte erläutern: Gesamtbetrag seit Diagnose in etwa (€)  -99 = keine Antwort | 1 = I had no additional expenses.  2 = I had additional expenses, please explain: Total amount since diagnosis approximately (€)  -99 = no answer |

**S4: Scoring procedure**

The instrument covers three dimension of subjective financial distress: 1) Financial worries; 2) Dissatisfaction across various life domains and 3) Challenging experiences with authorities and benefit providers (e.g., employment agency, health insurance). These dimensions are informed by qualitative prestudies and supported by factor analysis. Unweighted mean scores can be calculated for the subscales covering financial worries and challenging experiences with authorities. Regarding dissatisfaction across various life areas, it is necessary to reverse the polarity of the items 5, 7, 9, 11 and 13. Subsequently, the scores for each life domain's two questions should be multiplied, and an unweighted mean score including all life domains calculated. Each subscale can be used individually. Lower values are associated with low subjective financial distress, while higher values indicate higher subjective financial distress.

**S5: CFA Results: Individual subjective financial distress: Financial worries and Dissatisfaction across various life domains**

| **Nr.** | **Item** | **Standardized factor loadings (n=256)** | | |
| --- | --- | --- | --- | --- |
|  |  | **Dissatisfaction across various life domains** | | **Financial worries** |
| 1 | Ich mache mir Sorgen darüber, wie ich mit meiner Krebserkrankung mein Leben finanzieren soll. |  | | 0.96 |
| 2 | Ich mache mir Sorgen darüber, wie ich die Ausgaben aufgrund der Krebserkrankung decken kann. |  | | 0.94 |
| 3 | Ich mache mir Sorgen um die finanzielle Absicherung meiner Angehörigen. |  | | 0.81 |
| 4 | Ich mache mir Sorgen, dass ich zu wenig Geld für meine Zukunft zurücklegen kann. |  | | 0.71 |
| 5 | Wie zufrieden sind Sie mit Ihren finanziellen Möglichkeiten, eine gesunde Lebensweise zu realisieren? (-)  * Wie stark fühlen sie sich dadurch belastet? | 0.55 | |  |
| 6 | Wie zufrieden sind Sie mit Ihren finanziellen Möglichkeiten, Ihren Urlaub zu gestalten (-)  * Wie stark fühlen sie sich dadurch belastet? | 0.61 | |  |
| 7 | Wie zufrieden sind Sie mit Ihren finanziellen Möglichkeiten, Ihre Freizeit zu gestalten (-)  * Wie stark fühlen sie sich dadurch belastet? | 0.74 | |  |
| 8 | Wie zufrieden sind Sie mit Ihren finanziellen Möglichkeiten zur Gestaltung Ihrer Wohnsituation (-)  * Wie stark fühlen sie sich dadurch belastet? | 0.58 | |  |
| 9 | Wie zufrieden sind Sie mit Ihren finanziellen Möglichkeiten, Ihre Beziehungen zu Freunden und Familie zu gestalten? (-)* Wie stark fühlen sie sich dadurch belastet? | 0.76 | |  |
| **Model:** | |  |  | |
| Factor correlation  CMIN (df) | | 0.65, p < 0.001  889.303 (36) p < 0.001 | | |
| RMSEA | | 0.078, p < 0.05 | | |
| CFI | | 0.95 | | |
| TLI | | 0.93 | | |

**S6: CFA Results: Institutional subjective financial distress: Challenging experiences with authorities and benefit providers and counselling services**

| Nr. | Item | **Standardized factor loadings (*N*=256)** | |
| --- | --- | --- | --- |
|  |  | **Challenging experiences with authorities and benefit providers** | **Challenging Experiences with counseling services** |
| 1 | Ich fühle mich unter Druck gesetzt, Entscheidungen schnell zu treffen. | 0.62 |  |
| 2 | Ich habe Angst, dass ich Formulare oder Anträge falsch ausfülle. | 0.78 |  |
| 3 | Ich fühle mich durch lange Wartezeiten bei der Bearbeitung von Anträgen verunsichert. | 0.91 |  |
| 4 | Ich habe Angst, dass mir finanzielle Nachteile entstehen. | 0.78 |  |
| 5 | Ich empfinde die Kommunikation zwischen verschiedenen Stellen als unzureichend. | 0.73 |  |
| 6 | Ich fühle mich schlecht beraten. |  | 0.57 |
| 7 | Ich fühle mich unter Druck gesetzt, Entscheidungen schnell zu treffen. |  | 0.73 |
| 8 | Ich fühle mich durch lange Wartezeiten verunsichert. |  | 0.85 |
| 9 | Ich empfinde die Kommunikation zwischen verschiedenen Stellen als unzureichend. |  | 0.78 |
| **Model:** | |  |  |
| Factor correlation  CMIN (df) | | 0.2, p < 0.001  649.406 (36) p < 0.001 | |
| RMSEA | | 0.082 p < 0.05 | |
| CFI | | 0.93 | |
| TLI | | 0.91 | |

**S7: Item Statistics for Final PROM at T2 (N, Means, Standard Deviations, Skewness, Kurtosis, and Discriminatory Power of Items)**

| **No.** | **Item** | **N** | **Mean** | **SD** | **Skewness (SE)** | **Excess Kurtosis (SE)** | **Item-scale-correlation (corrected)** |
| --- | --- | --- | --- | --- | --- | --- | --- |
| 1 | Ich mache mir Sorgen darüber, wie ich mit meiner Krebserkrankung mein Leben finanzieren soll. | 255 | 2.28 | 1.29 | 0.80 (0.15) | -0.38 (0.30) | 0.88 |
| 2 | Ich mache mir Sorgen darüber, wie ich die Ausgaben aufgrund der Krebserkrankung decken kann. | 254 | 2.19 | 1.23 | 0.86 (0.15) | -0.25 (0.30) | 0.86 |
| 3 | Ich mache mir Sorgen um die finanzielle Absicherung meiner Angehörigen *(Kinder oder andere Familienmitglieder)* | 253 | 2.26 | 1.31 | 0.80 (0.15) | -0.51 (0.31) | 0.70 |
| 4 | Ich mache mir Sorgen, dass ich zu wenig Geld für meine Zukunft zurücklegen kann. | 255 | 2.65 | 1.47 | 0.32 (0.15) | -1.33 (0.30) | 0.80 |
| 5 | Wie zufrieden sind Sie mit Ihren finanziellen Möglichkeiten, eine gesunde Lebensweise zu realisieren? | 257 | 2.57 | 1.17 | 0.27 (0.15) | -0.85 (0.30) | - |
| 6 | Wie stark fühlen sie sich dadurch belastet? | 197 | 2.77 | 0.07 | 0.08 (0.17) | -0.29 (0.35) | - |
| 7 | Wie zufrieden sind Sie mit Ihren finanziellen Möglichkeiten, Ihren Urlaub zu gestalten? | 252 | 2.77 | 1.33 | 0.17 (0.15) | -1.17 (0.31) | - |
| 8 | Wie stark fühlen sie sich dadurch belastet? | 193 | 2.90 | 0.08 | 0.22 (0.18) | -0.40 (0.35) | - |
| 9 | Wie zufrieden sind Sie mit Ihren finanziellen Möglichkeiten, Ihre Freizeit zu gestalten? | 254 | 3.59 | 1.22 | -0.50 (0.15) | -0.69 (0.30) | - |
| 10 | Wie stark fühlen sie sich dadurch belastet? | 178 | 2.70 | 0.07 | 0.32 (0.18) | 0.05 (0.36) | - |
| 11 | Wie zufrieden sind Sie mit Ihren finanziellen Möglichkeiten zur Gestaltung Ihrer Wohnsituation? | 252 | 2.06 | 1.13 | 0.91 (0.15) | 0.03 (0.31) | - |
| 12 | Wie stark fühlen sie sich dadurch belastet? | 146 | 2.65 | 0.09 | 0.51 (0.20) | -0.16 (0.40) | - |
| 13 | Wie zufrieden sind Sie mit Ihren finanziellen Möglichkeiten, Ihre Beziehungen zu Freunden und Familie zu gestalten? | 254 | 2.18 | 1.12 | 0.64 (0.15) | -0.48 (0.30) | - |
| 14 | Wie stark fühlen sie sich dadurch belastet? | 160 | 2.75 | 0.08 | 0.30 (0.19) | -0.25 (0.38) | - |
| 15 | Ich fühle mich unter Druck gesetzt, Entscheidungen schnell zu treffen | 208 | 1.92 | 1.25 | 1.29 (0.17) | 0.57 (0.34) | 0.59 |
| 16 | Ich habe Angst, dass ich Formulare oder Anträge falsch ausfülle. | 223 | 2.22 | 1.30 | 0.83 (0.16) | -0.48 (0.32) | 0.67 |
| 17 | Ich fühle mich durch lange Wartezeiten bei der Bearbeitung von Anträgen verunsichert. | 217 | 2.48 | 1.35 | 0.48 (0.17) | -1.00 (0.33) | 0.82 |
| 18 | Ich habe Angst, dass mir finanzielle Nachteile entstehen. | 219 | 2.68 | 1.47 | 0.35 (0.16) | -1.25 (0.33) | 0.76 |
| 19 | Ich empfinde die Kommunikation zwischen verschiedenen Stellen als unzureichend. | 214 | 2.89 | 1.44 | 0.07 (0.17) | -1.33 (0.33) | 0.68 |

*Note. Deviations in N due to data missing; Polarity of item values was reversed for negatively poled items 5, 7, 9, 11, 13; Deviations in N for ratings of burden due to filter guidance: burden in the specified life area was not assessed if satisfaction was rated as 5 = very satisfied.*

**S8: Reliability of FIAT subscales**

| **Subscale** | **Number of items** | **Composite Reliability (Raykov´s Rho) (*N*)** | **Retest-Reliability r (*N*)** |
| --- | --- | --- | --- |
| **Financial worries** | 4 | 0.91 (255) | 0.75*** (60) |
| **Dissatisfaction across various life domains** | 10 | 0.96 (257) | 0.66*** (61) |
| **Challenging experiences with**  **authorities and benefit providers** | 5 | 0.88 (225) | 0.64*** (52) |

*Note. Deviations in N due to data missing; *p < 0.05, **p < 0.01, ***p < 0.001*

**S9: Correlations determining convergent construct validity**

| **FIAT Scales** | **Financial distress (EORTC-QLQ-C30, Q28)** | **Financial worries**  **(WDQ)** | **General distress (NCCN Distress Thermometer)** | **Burden due to uncertainties**  **(SCI)** |
| --- | --- | --- | --- | --- |
| **Financial**  **worries** | .57 *** | .85 *** | .44 *** | .63 *** |
| **N** | 253 | 243 | 229 | 211 |
| **Dissatisfaction across various life domains** | .61 *** | .73 *** | .47 *** | .53 *** |
| **N** | 255 | 241 | 229 | 213 |
| **Challenging experiences with**  **authorities and benefit providers** | .47 *** | .59 *** | .37 *** | .54 *** |
| **N** | 223 | 210 | 199 | 182 |

*Note. Deviations in N due to data missing; *p < 0.05, **p < 0.01, ***p < 0.001*

**S10: Correlations determining divergent construct validity**

|  | **Social Desirability (BIDR)** | **Conscientiousness**  **(Big 5)** | **Openness**  **(Big 5)** | **Agreeableness**  **(Big 5)** | **Extraversion**  **(Big 5)** | **Neuroticism**  **(Big 5)** |
| --- | --- | --- | --- | --- | --- | --- |
| **Financial worries**  **N** | -.06  236 | 0.00  238 | -.07  238 | -.02  238 | .02  235 | .16*  236 |
| **Dissatisfaction across various life domains**  **N** | .10  237 | -.05  237 | -.11  237 | -.01  237 | -.02  235 | .12  236 |
| **Challenging experiences with**  **authorities and benefit providers**  **N** | .02  208 | -.12  207 | -.09  207 | -.12  207 | -.19*  205 | .21*  206 |

*Note. Deviations in N due to data missing; *p < 0.05, **p < 0.01, ***p < 0.001*

**S11: Correlations determining criterion validity**

| **FIAT Scales** | **Health-related quality of life**  **(Q29 and Q30, EORTC-QLQ-C30)** | **Depression**  **(PHQ-4)** | **Anxiety**  **(PHQ-4)** |
| --- | --- | --- | --- |
| **Financial worries** | -.21*** | .38*** | .36*** |
| **N** | 255 | 243 | 241 |
| **Dissatisfaction across various life domains** | -.32*** | .46*** | .40*** |
| **N** | 257 | 242 | 240 |
| **Challenging experiences with**  **authorities and benefit providers** | -.27*** | .35*** | .27*** |
| **N** | 225 | 211 | 209 |

*Note. Deviations in N due to data missing; *p < 0.05, **p < 0.01, ***p < 0.001*

**S12: Results of linear regression analysis with risk factors potentially predicting Financial worries**

| **Potential risk factors for Financial worries (FIAT Subscale)** | **Standar-dized Beta- coefficient** | **T** | **Sig.** |
| --- | --- | --- | --- |
| Age | -0.11 | -1.11 | 0.27 |
| Gender female (Ref. male) | 0.03 | 0.30 | 0.76 |
| Insurance status statutory health insurance (Ref. private health insurance) | 0.12 | 1.38 | 0.17 |
| Living alone (Ref. living with partner) | -0.07 | -0.74 | 0.46 |
| Educational attainment | -0.14 | -1.42 | 0.16 |
| Personal net income before diagnosis | 0.10 | 0.63 | 0.53 |
| Personal net income after diagnoses | -0.08 | -0.51 | 0.61 |
| Loss of income (Ref. income didn´t change or increased) | 0.28 | 2.65 | 0.01 |
| Dependency of others on one´s personal income (Ref. No dependency of others one´s personal income) | 0.10 | 1.09 | 0.28 |
| Subjective socioeconomic status (subjective class identification) | -0.22 | -2.45 | 0.02 |
| Total Out-of-pocket-payments due to cancer diagnosis and treatment | -0.03 | -0.33 | 0.75 |
| **Model:** |  |  |  |
| F (df)  Sig. | 3.81 (11)  p < 0.001 | |  |
| Corrected R^2 | 0.21 |  |  |
